# Chloroplast genome of *Gaura parviflora* Douglas and its comparative analysis

**DOI:** 10.1080/23802359.2021.1878960

**Published:** 2021-03-11

**Authors:** Bin Zhang, Ruige Wu, Meng Liu, Xiaoran Cai, Yueqin Cheng

**Affiliations:** a College of plant protection, Henan Agricultural University, Zhengzhou, China; b Nanyang Grain and Oil Quality Inspection Center, Nanyang, China

**Keywords:** *Gaura parviflora* Douglas, chloroplast genome, comparative analysis, phylogenetic analysis

## Abstract

*Gaura parviflora* Douglas (Onagraceae) is an annual or perennial herbaceous plant from the prairie of North America. It has become a harmful exotic invading plant in China due to its strong adaptability, fast growth, massive propagation and reproduction. The complete chloroplast (cp) genome of *G. parviflora* was reported in this study. The size of the complete cp genome of *G. parviflora* is 161,318 bp in length, including a pair of inverted repeat (IR) regions of 27,402 bp, a large single-copy (LSC) region of 89,132 bp, and a small single-copy (SSC) region of 17,382 bp. A total of 130 genes were annotated, including 85 protein-coding genes, 37 tRNA genes, and 8 rRNA genes. Comparison of cp genomes of four species from Onagraceae indicated that Onagraceae cp genomes had high nucleotide diversity. In addition, a few highly variable regions of these cp genomes were also detected. The phylogenetic tree showed that *G. parviflora* is closely related to *Oenothera.* Thus, the chloroplast genome of *G. parviflora* can provide valuable genetic information for species identification and phylogenetic analysis.

*Gaura parviflora* Douglas ex Lehm.(synonymous name: *Oenothera curtiflora* W.L. Wagner & Hoch) is an annual or perennial herbaceous plant of the *Gaura* genus (Onagraceae) that is native to central and eastern North America and has been naturalized in Japan and distributed widely in Australia and South America (Chen et al. 2007). In recent years, it has been spreading rapidly in middle and eastern China. In the wild, dense single superior communities of *G. parviflora* are often formed due to their strong adaptability, fast growth and mass propagation (Du et al. 2003). Thus, *G. parviflora* has become a harmful exotic invading plant in China. In this study, we first reported the complete chloroplast genomes of *G. parviflora* based on Illumina HiSeq pair-end sequencing data to provide genetic information for further study about its spread process and evolutionary history.

Fresh leaves of *G. parviflora* used in this study were collected from Zhengzhou (34°48′3″N, 113°48′44″E), Henan Province, China. The specimen (ONAG20190012) was deposited in the herbarium of Henan Agricultural University. Total genomic DNA was extracted from fresh leaves using the modified CTAB protocol (Doyle and Doyle 1987). A short-insert library was prepared and sequenced on the Illumina HiSeq platform. The high-quality data obtained from the raw data were assembled using NOVOPlasy-v3.3 software (Dierckxsens et al. 2017) with *Epilobium ulleungensis* (GenBank accession number: NC_039575) as a reference. The complete cp genome was annotated using GeSeq (Tillich et al. 2017), and the annotation was corrected with Geneious Prime (Kearse et al. 2012). Finally, the complete cp genome of *G. parviflora* was submitted to GenBank (accession number: MT726052).

The size of the complete cp genome of *G. parviflora* is 161,318 bp, and the GC content of the whole plastid genome is 38.6%. It has a typical quadripartite construction, which contains a pair of inverted repeat (IR) regions of 27,402 bp, a large single-copy (LSC) region of 89,132 bp, and a small single-copy (SSC) region of 17,382 bp. The IR regions had a higher GC (43.1%) content than the LSC (36.7%) and SSC (34.0%) regions. A total of 130 genes were annotated, including 85 protein-coding genes, 37 tRNA genes, and eight rRNA genes. Among these genes, seven protein-coding genes, six tRNA genes and four rRNA genes are duplicated in the IR regions. Six tRNA genes and nine protein-coding genes contain a single intron, and two genes (*ycf3* and *clpP*) contain two introns.

We downloaded the chloroplast genomes of three other species (*Oenothera villaricae*, *E. ulleungensis* and *Ludwigia octovalvis*) in Onagraceae from the NCBI and compared them with the cp genome of *G. parviflora* using Geneious. DnaSP version 5.10.01 was used to calculate the nucleotide diversity (Pi) of these cp genome sequences. A high level of cp genomic nucleotide diversity (Pi = 0.0555) was detected in Onagraceae, which was higher than that in some other families, such as Malvaceae (Pi = 0.0250) and Paulowniaceae (Pi = 0.0007) (Li et al. 2020; Wang et al. 2021). The SSC region was the most variable, with the highest Pi value of 0.1070; IR regions were the most conserved, with Pi values of 0.0325; and the LSC region showed a medium Pi value of 0.0574. In the noncoding regions, the greatest variability was detected in the *trnH*-*psbA*, *rps12*-*clpP* and *rbcL*-*accD* regions, with Pi > 0.2303. In the coding regions, three genes, *clpP*, *ycf1* and *accD*, showed high levels of sequence variation (Pi > 0.1800), while 15 other genes had no base mutations. These hotspot regions could be used as potential markers for species identification in the future.

To determine the phylogenetic position of *G. parviflora* in Onagraceae, a dataset of 70 chloroplast coding regions from 13 species, including 11 Onagraceae species and two outgroup species (*Lagerstroemia speciosa* and *Duabanga grandiflora*), was aligned by using the MAFFT method in Geneious Prime (Kearse et al. 2012). A phylogenetic tree was then constructed using the maximum likelihood (ML) method on the CIPRES cluster (Miller et al. 2010). The ML phylogenetic results indicated that 11 species from Onagraceae were divided into two branches with high support (100%), *L. octovalvis* was a separate branch, and 10 other species made up the other branch. In the later branch, *E. ulleungensis* was the earliest species to diverge, and all eight *Oenothera* species clustered into a clade, which was sister to *G. parviflora* with 100% bootstrap support (Figure 1).

**Figure 1.**
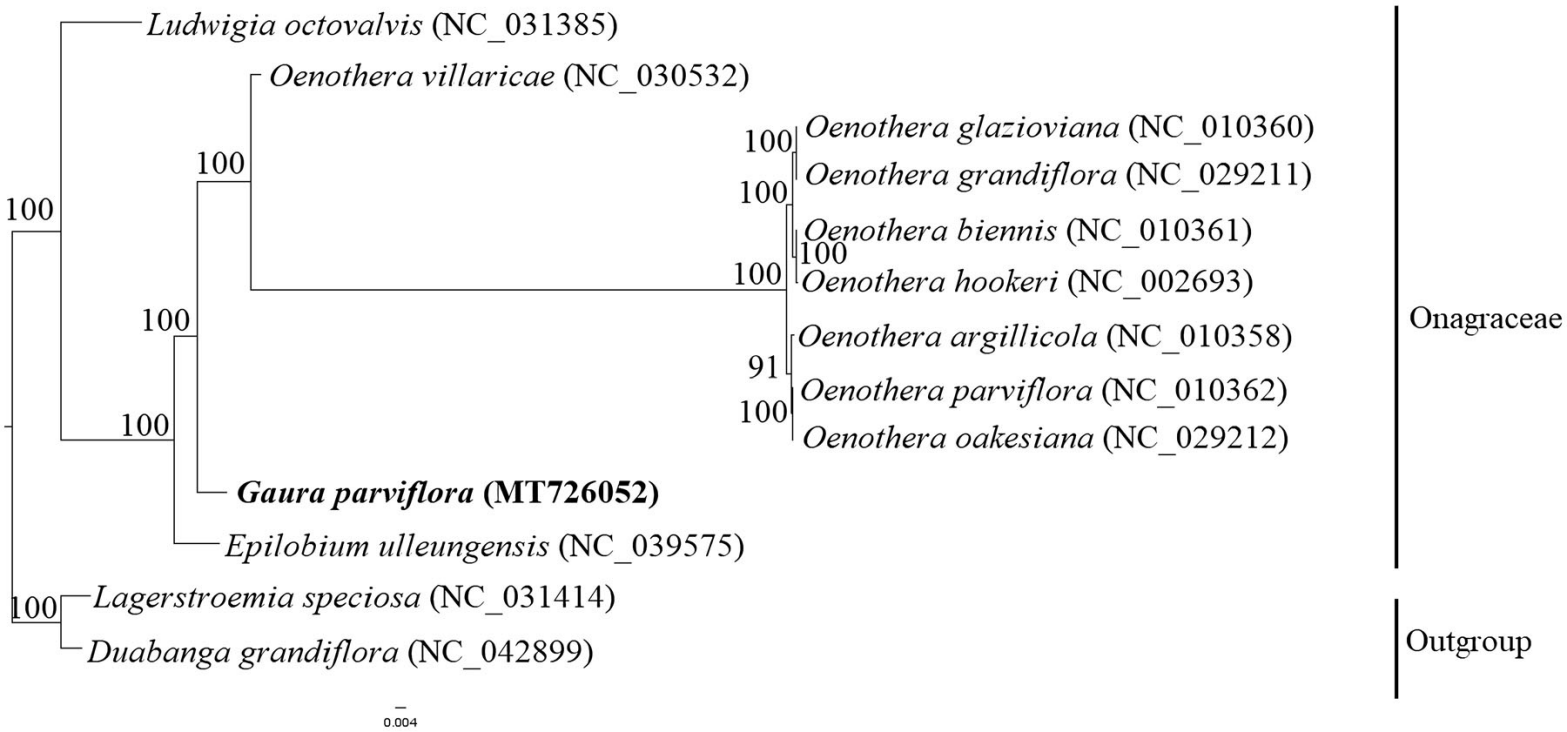
ML phylogenetic tree based on a dataset of 70 chloroplast coding regions. The numbers beside the node show bootstrap support values.

## Data Availability

The genome sequence data that support the findings of this study are openly available in GenBank of NCBI at (https://www.ncbi.nlm.nih.gov/) under the accession no. MT726052. The associated BioProject, SRA, and Bio-Sample numbers are PRJNA681490, SRR13171162, and SAMN16954857 respectively.
